# ‘Need-to-Know’ emergency medicine articles of 2014

**DOI:** 10.1186/s12245-015-0055-6

**Published:** 2015-03-07

**Authors:** Maite A Huis in ‘t Veld, Tu C Nguyen, Joseph P Martinez, Amal Mattu

**Affiliations:** Emergency Medicine Residency, University of Maryland Medical Center, Baltimore, Maryland USA; Department of Emergency Medicine, University of Maryland School of Medicine, 110 South Paca Street, 6th Floor, Suite 200, Baltimore, MD 21201 USA

**Keywords:** Literature, Update, Sepsis, Shock, Resuscitation, Cardiac, Arrest, Airway, Defibrillation, Hypotension

## Abstract

Every year, thousands of articles are published in numerous medical journals that relate to the clinical practice of medicine. However, it is impossible for a single clinician to stay abreast of the literature, let alone to determine which articles should change daily practice. Physicians in our department have searched the emergency medicine and the specialty literature of 2014 to determine which articles are most relevant to the clinical practice of emergency medicine, summarized them, and listed key take-home points from these ‘need-to-know’ articles.

## Review

### Introduction

Since 2004, our faculty has made a concerted effort on an annual basis to cull through the medical literature in order to pick out articles that are relevant to acute care providers. The articles are chosen based on the presence of useful ‘take-home points’ that can influence the clinical practice of acute care providers and improve patient care. Once the articles are chosen, they are organized by subspecialty area and are briefly summarized with key information about the study and a few take-home points. In this year, we have once again collected our favorite articles and we hereby present our top picks of 2014, summarized and with practical take-home points, for the readers of *International Journal of Emergency Medicine*.

We’ve chosen to present the summaries in bulleted format, and each summary concludes with a take-home point for ease of reading. A detailed review of the methodology and statistics was not included, in the interest of brevity. However, we encourage readers that are interested in this information to read the full manuscripts. We sincerely hope you enjoy this summary of the ‘need-to-know’ emergency articles of 2014!

### Airway

Cohen L, Athaide V, Wickham ME, et al. The effect of ketamine on intracranial and cerebral perfusion pressure and health outcomes: a systematic review. *Ann Emerg Med* 2015, 65:43-51.

- This review of ten studies compared ketamine with other intravenous induction agents in regard to intracranial and cerebral perfusion pressures, neurologic outcomes, ICU length of stay, and mortality.
- Two studies reported a brief increase in intracranial pressure after administration of ketamine but no evidence of sustained changes in intracranial pressure or cerebral perfusion pressure. None of the studies reported significant differences in cerebral perfusion pressure, neurologic outcomes, ICU length of stay, or mortality rate.
- Take-home point: This review suggests that, compared with other intravenous induction agents, the use of ketamine in critically ill patients does not adversely affect intracranial pressure, neurologic outcomes, or mortality rate.

McMullan J, Gerecht R, Bonomo J, et al. Airway management and out-of-hospital cardiac arrest outcome in the CARES registry. *Resuscitation* 2014, 85(5):617-622.

- A retrospective analysis of the CARES registry (12,875 out-of-hospital cardiac arrests (OHCAs)) revealed that patients who did not receive an advanced airway during initial resuscitation had a higher likelihood for hospital admission (OR = 1.31; 95% CI = 1.16 to 1.49), hospital survival (OR = 2.96; 95% CI = 2.50 to 3.51), and hospital discharge with good neurologic outcome (OR = 4.24; 95% CI = 3.46 to 5.20) compared with those who received advanced airways.
- Among patients who received advanced airways, endotracheal intubation was associated with better outcomes than use of a supraglottic airway.
- Take-home point: In OHCA, airway management should focus on proper bag-valve-mask ventilation, not insertion of an advanced airway.

Kim WY, Kwak MK, Ko BS, et al. Factors associated with the occurrence of cardiac arrest after emergency tracheal intubation in the emergency department. *PLoS ONE* 2014, 9:e112779.

- This retrospective analysis revealed that nearly 2% of all non-traumatic emergency department intubations were associated with cardiac arrest within 10 min following intubation.
- The most important, statistically significant predictor for post-intubation cardiac arrest was systolic hypotension (systolic blood pressure <90 mmHg). The most common initial arrest rhythm was pulseless electrical activity (PEA), suggesting that the cause of arrest was not a primary cardiac event.
- The most likely presumed cause of PEA was an abrupt loss of preload and consequent critical hypotension in response to positive-pressure ventilation.
- Take-home point: Because of the observed risk of hemodynamic collapse after intubation, the authors question whether pre-intubation hypotension in critically ill patients should be treated with aggressive fluid resuscitation and/or vasopressors. Though randomized studies to evaluate this proposal have not yet been done, it seems logical to optimize preload in critically ill patients before tracheal intubation whenever possible and to have vasopressors readily available in the event of a post-intubation drop in blood pressure.

### Cardiology

Littmann L, Bustin DJ, Haley MW. A simplified and structured teaching tool for the evaluation and management of pulseless electrical activity. *Med Princ Pract* 2014, 23:1-6.

- This article proposes a novel approach that simplifies the differential diagnosis, evaluation, and management of patients with PEA, using only electrocardiography and bedside ultrasonography. In this approach, PEA arrest is classified as wide or narrow QRS complex.
- A narrow complex on the ECG suggests that a mechanical problem is impairing right ventricular inflow or outflow (tamponade, tension pneumothorax, mechanical hyperinflation, and pulmonary embolism). Ultrasound will show a hyperdynamic left ventricle (a ‘pseudo PEA’). Treatment should be based on ultrasound findings, and IV fluids should be administered. Chest compressions should be used with caution, if at all, in these patients, because it might further impair mechanical function.
- A wide complex on the ECG suggests a metabolic problem (hyperkalemia and sodium channel blocker toxicity). If hyperkalemia is suspected, sodium bicarbonate and calcium should be administered. If sodium channel blocker toxicity is suspected, sodium bicarbonate boluses should be given.
- Take-home point: Consider using this simple new approach for patients in PEA arrest. Validation studies are needed.

Waldo S, Brenner DA, Li S, et al. Reperfusion times and in-hospital outcomes among patients with an isolated posterior myocardial infarction. *Am Heart J* 2014, 167:350-354.

- In the NCDR-ACTION-GWTG registry (with 117,739 records), isolated posterior ST-elevation myocardial infarction (STEMI) patients received percutaneous coronary intervention (PCI) less often and later than those with non-posterior STEMI.
- Patients with isolated posterior STEMI had a greater risk of in-hospital cardiac arrest, cardiogenic shock, and death.
- Take-home point: Posterior STEMI should be strongly considered whenever ST-segment depression is noted in the right precordial leads. In this scenario, repeating the ECG with posterior leads can help to confirm the diagnosis.

Lemkin DL, Witting MD, Allison MG, et al. Electrical exposure risk associated with hands-on defibrillation. *Resuscitation* 2014, 85:1330-1336.

- Hands-on defibrillation during cardiopulmonary resuscitation has been proposed as a technique that will minimize interruptions in chest compressions. However, this practice introduces concerns about the safety of the people who remain in contact with the patient when the shock impulses are delivered. In this cadaver study, the authors sought to determine the worst-case energy transfer that the rescuers might encounter while performing hands-on defibrillation.
- Six cadavers were exposed to defibrillation voltages while attached to a multipoint measurement circuit. Voltage was measured on different surfaces of the bodies. Using a formula that integrated the voltage measurements, the investigators calculated a theoretic rescuer-received dose.
- All calculated rescuer-received doses exceeded the safety cutoff of 1 J.
- Take-home point: This study does not answer the question whether hands-on defibrillation is safe, but it does raise concern regarding the safety of care providers.

Dumas F, Bougouin W, Geri G, et al. Is epinephrine during cardiac arrest associated with worse outcomes in resuscitated patients? *J Am Coll Cardiol* 2014; 64(22):2360-2367.

- The utility of epinephrine in the setting of cardiac arrest has been the subject of debate in recent years. This retrospective, observational cohort study sought to investigate the relationship between prehospital use of epinephrine and functional survival among patients with out-of-hospital cardiac arrest (OHCA).
- Survival and survival with good neurologic outcome were less likely among those who received epinephrine than among those who did not receive epinephrine. Higher cumulative doses of epinephrine were linearly associated with worse outcomes compared with lower doses of epinephrine.
- The use of therapeutic hypothermia and early cardiac catheterization did not appear to mitigate the adverse effects of epinephrine.
- Take-home point: This article does not provide a final answer to the role of epinephrine in cardiac arrest; however, it does suggest that the use of epinephrine for OHCA in the prehospital setting could be harmful and that higher cumulative doses are associated with adverse neurologic outcomes.

### Gastroenterology

Kim JJ, Tsukamoto MM, Mathur AK, et al. Delayed paracentesis is associated with increased in-hospital mortality in patients with spontaneous bacterial peritonitis. *Am J Gastroenterol* 2014; 109:1436-1442.

- This retrospective study of consecutive patients with spontaneous bacterial peritonitis (SBP) was compared with the outcomes of those who received early paracentesis (EP; <12 h) and those who received delayed paracentesis (DP; 12 to 72 h).
- Patients in the DP group had higher in-hospital mortality (27% vs. 13%, *p* = 0.007), longer ICU and hospital stays, and a higher 3-month mortality rate.
- Each hour of delay in paracentesis was associated with 3.3% increase in the in-hospital mortality rate.
- Take-home point: Perform paracentesis on all patients being admitted with suspected SBP and do not delay paracentesis until after admission to an inpatient bed.

Rahimi RS, Singal AG, Cuthbert JA, et al. Lactulose vs polyethylene glycol 3350-electrolyte solution for treatment of overt hepatic encephalopathy: the HELP randomized clinical trial. *JAMA Intern Med* 2014; 174(11):1727-1733.

- This randomized trial compared the standard treatment (lactulose) and the use of polyethylene glycol 3350-electrolyte solution (PEG [normally used for colonoscopy preparation]) for patients with overt hepatic encephalopathy.
- The primary outcome measurement was improvement in the hepatic encephalopathy grade of one level or more (using the hepatic encephalopathy scoring algorithm (HESA), in which 0 is normal and 4 is coma).
- This study involved only a small number of patients (25 in each arm), but the trend in the findings is telling: 91% of the patients in the PEG arm met the primary outcome compared with 52% in lactulose arm. Patients in the PEG arm had resolution of encephalopathy in 1 day compared with 2 days in the standard arm.
- Take-home point: PEG appears to be superior to lactulose for the treatment of hepatic encephalopathy.

### Hematology

Righini M, Van Es J, Den Exter PL, et al. Age-adjusted D-dimer cutoff levels to rule out pulmonary embolism: the ADJUST-PE study. *JAMA* 2014; 311:1117-1124.

- This multicenter, multinational, and prospective diagnostic management outcome study looked at the utility of an age-adjusted d-dimer level. For patients 50 years and older, the d-dimer level was considered positive only if it was higher than the patient’s age multiplied by 10.
- More than 3,000 patients were enrolled in this study. Patients’ pretest probability of pulmonary embolism (PE) was assessed using either Geneva or Wells criteria. Patients with high clinical probability were evaluated with CT scans and patients with low or intermediate clinical probability underwent d-dimer testing. Only those above the age-adjusted cutoff underwent CT scanning. The others were followed for 3 months.
- The failure rate for those patients with a d-dimer level above 500 μg/L but below the age-adjusted cutoff was 1/331 patients (0.3%). Among those 75 years or older, the age-adjusted cutoff increased the proportion that could have PE ruled out without further testing by fivefold.
- Take-home point: In patients older than 50 years, the use of an age-adjusted cutoff for d-dimer might allow more patients to be discharged safely without advanced testing.

Chatterjee S, Chakraborty A, Weinberg I, et al. Thrombolysis for pulmonary embolism and risk of all-cause mortality, major bleeding, and intracranial hemorrhage. *JAMA* 2014; 311:2414-2421.

- In a systematic literature review of randomized controlled trials, the authors compared mortality rates in groups receiving thrombolytics and groups treated with anticoagulants alone.
- Patients who received thrombolytics had a lower all-cause mortality rate (2.17% vs. 3.89%; *p* = 0.01), with a number needed to treat of 59. This mortality benefit was preserved when the subset of intermediate-risk PE was examined.
- This benefit came at the expense of more major bleeding (9.24% vs. 3.42% (*p* < 0.001); NNH = 18) and more intracranial hemorrhage (1.46% vs. 0.19% (*p* = 0.002); NNH = 78).
- Importantly, the risk of major bleeding was significantly increased only in the subset of patients older than 65 years.
- Take-home point: Thrombolysis for PE is associated with lower all-cause mortality compared with anticoagulation alone, but it comes with the risk of increased major bleeding, especially in patients over the age of 65 years.

Meyer G, Vicaut E, Danays T, et al. Fibrinolysis for patients with intermediate-risk pulmonary embolism. *N Engl J Med* 2014; 370:1402-1411.

- The Pulmonary Embolism Thrombolysis (PEITHO) trial was a randomized, double-blind study that compared the administration of tenecteplase + heparin with the administration of placebo + heparin to patients with intermediate-risk pulmonary emboli. ‘Intermediate risk’ referred to pulmonary emboli in patients who were hemodynamically stable but had evidence of right ventricular dysfunction by echocardiography or computed tomography (CT) as well as elevated troponin values.
- The primary outcomes of death from any cause or hemodynamic decompensation or collapse within 7 days occurred in 2.6% of the tenecteplase group compared with 5.6% of the placebo group (*p* = 0.02).
- The risks of major hemorrhage and stroke were significantly higher in the tenecteplase group than in the placebo group: 6.3% vs. 1.2% for hemorrhage (*p* < 0.001) and 2.4% vs. 0.2% for stroke (*p* = 0.003), respectively.
- Take-home point: Fibrinolytic therapy might prevent hemodynamic decompensation in patients with intermediate-risk pulmonary embolism, but this benefit comes at the cost of significantly higher major bleeding and stroke.

### Infectious diseases

Asfar P, Teboul JL, Radermacher P. High versus low blood-pressure target in patients with septic shock. *N Engl J Med* 2014;370(17):1583-1593.

- The Surviving Sepsis Campaign recommends a target mean arterial pressure (MAP) of at least 65 mmHg; however, it is unclear whether this target is more or less effective than a higher target MAP.
- This randomized controlled trial assigned septic patients to a high target MAP goal (80 to 85 mmHg) or to a lower target MAP (65 to 70 mmHg).
- Patients who were randomized to the high target MAP goal required less renal replacement therapy, but no difference in mortality was found.
- Take-home point: These findings suggest that the traditional MAP goal of 65 mmHg is reasonable in septic patients, as higher targets do not appear to be associated with improvements in mortality.

ProCESS Investigators. A randomized trial of protocol-based care for early septic shock. *N Engl J Med* 2014; 370:1683-1693.

- Early goal-directed therapy (EGDT) for patients presenting to the emergency department with sepsis has been widely implemented since the Rivers study was published in 2001. The multicenter, prospective ProCESS trial was designed to assess whether protocol-based resuscitation is superior to usual care. It also evaluated whether a protocol with central hemodynamic monitoring was superior to a protocol without it.
- No differences were observed between groups in regard to the mortality rate at 60 days, 90 days, or 1 year. No differences were observed in secondary endpoints (cardiovascular failure, respiratory failure, hospital length of stay, or discharge disposition). Acute renal failure rates were higher in the protocol-based standard therapy group.
- Take-home point: This study did not find a mortality or morbidity benefit for protocol-based EGDT, including treatment strategies involving central hemodynamic monitoring, in septic patients presenting to the emergency department compared with care that was provided according to the treating physician’s judgment.

The ARISE Investigators and the ANZICS Clinical Trials Group. Goal-directed resuscitation for patients with early septic shock. *N Engl J Med* 2014; 371:1496-1506.

- This Australian, multicenter trial randomized patients with early septic shock to an EGDT protocol including invasive central venous pressure monitoring or to usual care.
- EGDT did not reduce all-cause mortality at 90 days. There was also no significant difference in survival time, in-hospital mortality, duration of organ support, or length of hospital stay.
- Take-home point: Both the ARISE and ProCESS studies suggest that EGDT, including invasive central venous pressure monitoring and central venous oxygen saturation measurements, is not needed to guide therapy in patients in septic shock. The key elements in optimal patient care appear to be (1) early recognition of sepsis and initiation of broad-spectrum antibiotics, (2) aggressive fluid replacement, and (3) monitoring of perfusion with non-invasive methods such as serial lactate levels.

O’Horo JC, Maki DG, Krupp AE, et al. Arterial catheters as a source of bloodstream infection: a systematic review and meta-analysis. *Crit Care Med* 2014; 42:1334-1339.

- This systematic review concluded that arterial lines carry the same risk of catheter-related bloodstream infections as short-term central venous catheters. The risk of infection is higher with the femoral approach.
- Take-home point: Pay attention to sterile technique when placing an arterial line. The use of chlorhexidine-impregnated dressings might reduce the infection rate.

Peterson D, McLeod S, Woolfrey K, et al. Predictors of failure of empiric outpatient antibiotic therapy in emergency department patients with uncomplicated cellulitis. *Acad Emerg Med* 2014; 21:526-531.

- A prospective cohort of 598 ED patients was analyzed for risk factors associated with failure of outpatient treatment for cellulitis.
- Risk factors included fever >38°C at triage (OR = 4.3; 95% CI = 1.6 to 11.7), chronic leg ulcers (OR = 2.5; 95% CI = 1.1 to 5.2), chronic edema or lymphedema (OR = 2.5; 95% CI = 1.5 to 4.2), prior cellulitis in the same area (OR = 2.1; 95% CI = 1.3 to 3.5), and cellulitis at a wound site (OR = 1.9; 95% CI = 1.2 to 3.0).
- Take-home point: Patients with uncomplicated cellulitis and any of the above risk factors are at risk of failing outpatient treatment and should be considered for observation or admission for intravenous antibiotics.

Barnett ML, Linder JA. Antibiotic prescribing to adults with sore throat in the United States, 1997-2010. *JAMA Intern Med* 2014; 174:138-140.

- This research letter highlights the point that, although only 10% of sore throats in adults are caused by Group A *Streptococcus*, approximately 60% of patients with a sore throat are given antibiotics in the emergency department.
- The financial cost of prescribing unnecessary antibiotics to adults with sore throat in all ambulatory settings in the USA from 1997 to 2010 was $500 million.
- Penicillin, the appropriate first-line antibiotic, was prescribed only 9% of the time, whereas azithromycin and other less appropriate choices were prescribed more often.
- Take-home point: Be judicious about the use of antibiotics in adults with a sore throat. If antibiotics are to be used, choose appropriately.

Harbarth S, von Dach E, Pagani L, et al. Randomized non-inferiority trial to compare trimethoprim/sulfamethoxazole plus rifampicin versus linezolid for the treatment of MRSA infection. *J Antimicrob Chemother* 2015; 70:264-272.

- Linezolid has gained popularity for the treatment of methicillin-resistant Staphylococcus aureus (MRSA) infections.
- This randomized, single-center, non-inferiority trial of 150 patients with MRSA infection compared with trimethoprim/sulfamethoxazole plus rifampin versus linezolid. No significant difference was found in cure rates, adverse events, or serious adverse events between treatment groups. However, the generic combination was much cheaper than linezolid.
- Take-home point: Consider use of the generic combination of trimethoprim/sulfamethoxazole plus rifampin as a more economical alternative to the use of linezolid for MRSA infections.

### Neurology

Shahrami A, Assarzadegan F, Hatamabadi HR, et al. Comparison of therapeutic effects of magnesium sulfate vs. dexamethasone/metoclopramide on alleviating acute migraine headache. *J Emerg Med* 2015; 48:69-76.

- This randomized double-blind clinical trial of 70 patients with migraines demonstrated that magnesium (1 g in 100 cc NS IV) provided quicker relief than did combination therapy of metoclopramide/dexamethasone (8 mg/10 mg in 100 cc NS IV).
- Magnesium had fewer side effects than combination therapy, although the difference was not statistically significant.
- Take-home point: Consider magnesium when treating patients with migraines in the ED.

Hubner P, Meron G, Kurkciyan I, et al. Neurologic causes of cardiac arrest and outcomes. *J Emerg Med* 2014;47:660-667.

- This was a retrospective analysis of prospectively collected data from 154 patients presenting to the emergency department in cardiac arrest secondary to a neurologic disorder.
- Half of the patients presented with pulseless electrical activity, 40% had asystole, and 10% presented with ventricular fibrillation.
- The most common causes were subarachnoid hemorrhage (48%), intracerebral hemorrhage (21%), epileptic seizure (15%), and ischemic stroke (7%).
- Patients with cardiac arrest caused by subarachnoid hemorrhage rarely survive to hospital discharge.
- Take-home point: Neurologic catastrophes account for a small subset of the causes of cardiac arrest but are rarely associated with ventricular fibrillation. Subarachnoid hemorrhage is the most likely neurologic diagnosis in patients presenting in cardiac arrest and has a dismal prognosis.

He J, Zhang Y, Xu T, et al. Effects of immediate blood pressure reduction on death and major disability in patients with acute ischemic stroke: the CATIS randomized clinical trial. *JAMA* 2014; 311:479-489.

- The China Antihypertensive Trial in Acute Ischemic Stroke (CATIS) was a large, multicenter, randomized, controlled study of 4,071 adults with acute ischemic stroke and hypertension (SBP; 140 to 220 mmHg).
- Patients in the intervention arm were given antihypertensive medications, with the goal of lowering SBP by 10% to 25% within the first 24 h and achieving a goal of SBP <140 mmHg and DBP <90 mmHg within 7 days. The control arm had their home antihypertensive medications discontinued. The primary outcome was death or major disability (scores of 3 to 5 on the modified Rankin scale) within 14 days. Secondary outcome was death or major disability at 3 months.
- In the intervention group, blood pressure was significantly lower at 24 h and 14 days. However, there was no significant difference in the primary outcome (33.6% in the intervention group and 33.6% in the control group) or in the secondary outcome (25.2% vs. 25.3%).
- Take-home point: These findings suggest that, unless a patient with acute ischemic stroke is severely hypertensive (SBP >220 mmHg), antihypertensive therapy does not change the outcome. For patients who require blood pressure treatment for other reasons, it appears to be safe to initiate treatment.

### OB/GYN

Danielsson B, Wikner BN, Källén B. Use of ondansetron during pregnancy and congenital malformations in the infant. *Reprod Toxicol* 2014; 50:134-137.

- Data from the Swedish Medical Birth Register and Swedish Register of Prescribed Drugs (1998-2012) were used to identify infants exposed to maternal use of ondansetron and meclizine in the first trimester.
- The study involved 1,501,434 infants, 1,349 of whom had been exposed to ondansetron. There was no statistically significant increased risk for severe congenital malformations with use of ondansetron (OR = 1.11; 95% CI = 0.81 to 1.53) or meclizine (OR = 0.95; 95% CI = 0.90 to 1.00). However, the use of ondansetron during the first trimester showed an increased risk of cardiovascular defects (OR = 1.62; 95% CI = 1.04 to 2.14), particularly cardiac septum defects (OR = 2.05; 95% CI = 1.19 to 3.28). This risk was not present with use of meclizine (OR = 1.02; 95% CI = 0.92 to 1.12).
- This study is limited because its data were based on filled prescriptions (it is unclear if the patients actually took the prescribed medication).
- Take-home point: Even though this is a retrospective analysis with limitations, the data indicate an alarming increased risk of cardiac malformations with the use of ondansetron in the first trimester. Until larger prospective trials are available, it is advisable not to use ondansetron for treatment of nausea and emesis in the first trimester.

### Ophthalmology

Waldman N, Densie IL, Herbison P. Topical tetracaine used for 24 h is safe and rated highly effective by patients for the treatment of pain caused b*y* corneal abrasions: a double-blind, randomized clinical trial. *Acad Emerg Med* 2014; 21:374-382.

- This double-blind, prospective clinical trial randomized 116 ED patients with uncomplicated corneal abrasions to receive ophthalmic tetracaine or ophthalmic saline drops for 24 h. Primary outcomes were corneal healing at 48 h, 1-week, and 1-month symptoms, visual analog scale (VAS) pain scores, and complications.
- Ophthalmic tetracaine use for corneal abrasions showed no statistically significant advantage in healing of corneal abrasions, pain scale scores, or complications compared with normal saline drops. Patients who received tetracaine had higher overall satisfaction scores.
- Take-home point: The use of topical tetracaine in patients with uncomplicated corneal abrasions was not associated with a significant reduction of pain or symptom relief; however, it is a safe consideration and was associated with higher patient satisfaction scores.

### Orthopedics

Eskin B, Shih RD, Fiesseler FW, et al. Prednisone for emergency department low back pain: a randomized controlled trial. *J Emerg Med* 2014; 47:65-70.

- This prospective randomized controlled study involved 79 patients with uncomplicated lower back pain, who came to an ED within 2 days after sustaining a bending/twisting injury. At discharge, they were randomized to take either 50 mg of prednisone or a placebo for 5 days.
- After 5 days, patients were contacted to assess their pain level (none, mild, moderate, and severe) and activity level. There was no significant difference in pain or activity levels between the treatment arms. More patients in the prednisone group sought additional medical treatment (40% vs. 18%; 95% CI = 0 to 43).
- Take-home point: In this small study, prednisone provided no benefit to patients presenting with acute lower back pain after a twisting/bending injury.

Gorchynski J, Karabidian E, Sanchez M. The “‘syringe’” technique: a hands-free approach for the reduction of acute nontraumatic temporomandibular dislocations in the emergency department. *J Emerg Med* 2014; 47:676-681.

- The authors propose a novel technique for reduction of temporomandibular joint (TMJ) dislocation.
- This technique uses a syringe placed between the posterior molars. The patient or the physician rotates the syringe back and forth, which allows it to glide the anteriorly displaced condyle back into its normal anatomic position. Procedural sedation or intravenous analgesia is not required, and the physician does not need to insert his or her fingers into the patient’s mouth.
- In their small cohort (31 patients), the authors had a 97% success rate.
- Take-home point: This simple, novel technique seems promising for reduction of TMJ dislocation.

### Pediatrics

Chamberlain JM, Okada P, Holsti M, et al. Lorazepam vs diazepam for pediatric status epilepticus: a randomized clinical trial. *JAMA* 2014; 311:1652-1660.

- Lorazepam is not approved by the Food and Drug Administration (FDA) for use in pediatric status epilepticus, despite its potential advantages, which include improved effectiveness in terminating convulsions, a longer duration of action, and a lower incidence of respiratory depression.
- However, lorazepam is often used preferentially (despite the lack of formal approval) by many care providers who believe that it is more effective than diazepam in treating status epilepticus.
- This multi-center randomized controlled trial compared diazepam, 0.2 mg/kg, and lorazepam, 0.1 mg/kg, and found no statistically significant differences in cessation of convulsions at 10 min or need for assisted ventilation. Patients who received lorazepam had higher rates of sedation.
- Take home point: This study suggests that lorazepam is not superior to diazepam for the treatment of status epilepticus in pediatric patients.

Leeuwenburgh MM, Stockmann HB, Bouma WH, et al. A simple clinical decision rule to rule out appendicitis in patients with nondiagnostic ultrasound results. *Acad Emerg Med* 2014; 21:488-496.

- Pediatric patients suspected of having appendicitis despite a non-conclusive or negative ultrasound pose a diagnostic dilemma for the emergency physician.
- Based on a validation cohort, a proposed clinical decision rule was developed to identify patients who are safe for discharge with next-day follow-up. Patients were included in that category if fewer than two of the following predictors were present: male sex, migration of pain to the right lower quadrant, vomiting, and white blood cell count higher than 12.0 × 10^9^/L. The use of this decision rule reduced the probability of appendicitis from 20% to 6% in the validation arm of this study, with a negative predictive value of 94%.
- Take-home point: This clinical decision rule identifies pediatric patients with a very low likelihood of appendicitis. They can be considered appropriate candidates for discharge home with close observation.

### Toxicology

Chan BS, Buckley NA. Digoxin-specific antibody fragments in the treatment of digoxin toxicity. *Clin Toxicol* 2014;52:824-36.

- This was a systematic literature review of the use of digoxin-Fab between 1946 and 2013.
- Full neutralizing doses of digoxin-Fab are expensive and might not be required or necessary if the patient does not present with life-threatening toxicity or cardiac arrest. On the other hand, the administration of full neutralizing doses can be justified in cases of cardiac arrest associated with digoxin toxicity.
- To treat an acute ingestion, administer 40 to 80 mg (1 or 2 vials) and repeat the dose as necessary to achieve a clinical response. In chronic toxicity, administer 40 mg (1 vial) and titrate with slow infusion. If the patient does not respond within 60 min, repeat the dose.
- Take-home point: The use of digoxin-Fab is safe and justified in patients with life-threatening toxicity.

Mueller SW, Preslaski CR, Kiser TH, et al. A randomized, double-blind, placebo-controlled, dose range study of dexmedetomidine as adjunctive therapy for alcohol withdrawal. *Crit Care Med* 2014; 42:1131-1139.

- Twenty-four intensive care unit (ICU) patients with severe alcohol withdrawal were randomized to receive lorazepam + placebo or lorazepam + dexmedetomidine. The dexmedetomidine was given as a low dose (0.4 mcg/kg/h) or a high dose (1.2 mcg/kg/h).
- The dexmedetomidine group showed a statistically significant reduction in 24-h lorazepam requirement compared with the placebo group (−56 vs. −8 mg (*p* = 0.037)).
- There was no significant difference in the 7-day cumulative lorazepam requirement between the placebo and dexmedetomidine groups.
- Bradycardia occurred more frequently in the dexmedetomidine group: (4 versus 0 patients (the difference is not significant)). It was not reported if these patients required treatment for bradycardia.
- Take-home point: Dexmedetomidine reduced short-term benzodiazepine requirements but not long-term cumulative doses when a symptom-triggered approach was used. It is important to monitor patients for bradycardia, especially when using high doses of dexmedetomidine.

### Trauma

Slessor D, Hunter S. To be blunt: are we wasting our time? Emergency department thoracotomy following blunt trauma: a systematic review and meta-analysis. *Ann Emerg Med* 2014 Oct 23 [Epub ahead of print].

- This was a systematic review and meta-analysis of articles looking at out-of-hospital or ED thoracotomies for blunt trauma. It included 27 articles in the review and 13 in the meta-analysis.
- Of a total of 1,369 thoracotomies, 21 patients survived with good neurologic outcomes, a rate of 1.5%.
- All of the survivors had vital signs present at the scene or on admission. Of those survivors whose duration of CPR was recorded, it was <15 min in all cases.
- The authors propose an algorithm to aid in decision-making in cases of blunt traumatic arrest.
- Take-home point: Patients without signs of life at the scene, with prolonged CPR, or with head injury incompatible with good outcome should not be considered for ED thoracotomy. The procedure should be undertaken only if appropriately experienced and skilled staff members are available.

### Ultrasound

Smith-Bindman R, Aubin C, Bailitz J, et al. Ultrasonography versus computed tomography for suspected nephrolithiasis. *N Engl J Med* 2014; 371:1100-1110.

- In this large, multicenter study, patients with suspected nephrolithiasis were randomized to point-of-care ultrasound performed by the emergency physician, radiology ultrasound, or abdominal computed tomography. Additional imaging was performed at the discretion of the treating physician.
- Patients receiving point-of-care ultrasound had no significant difference in high-risk diagnoses or serious adverse events and had significantly lower cumulative radiation exposure.
- Even though ultrasound groups were at higher risk of additional diagnostic imaging, the total cost for their emergency department stay was still lower.
- Take-home point: These results do not suggest that ultrasonography should be the only imaging modality used for patient with suspected nephrolithiasis, but rather that point-of-care ultrasonography should be used as the initial diagnostic imaging test, with further imaging studies performed at the discretion of the physician. The use of ultrasound as initial screening test has the potential to decrease radiation exposure dramatically.

Al Deeb M, Barbic S, Featherstone R, et al. Point-of-care ultrasonography for the diagnosis of acute cardiogenic pulmonary edema in patients presenting with acute dyspnea: a systematic review and meta-analysis. *Acad Emerg Med* 2014; 21:843-852.

- This systematic review focused on patients with a moderate to high pretest probability for acute cardiogenic pulmonary edema (ACPE). An ultrasound study showing B-lines can be used to strengthen an emergency physician’s working diagnosis of ACPE. Pooled data revealed a sensitivity of 94.1%.
- Take-home point: In patients with a low pretest probability for ACPE, a negative US study almost excludes it. Pooled data revealed a specificity of 92.4%.

## Conclusion

It is critically important for acute care providers to remain up-to-date on the recent advances in the medical field that will improve patient care. Given the tremendous number of medical journals and research publications that are produced each year, keeping abreast of the literature is a daunting task. We hope that this publication has been helpful in updating acute care providers on the recent literature and will help with patient care.

